# Schizophrenia and OCD association, psychopharmacological intervention and cognitive-behavioral therapy

**DOI:** 10.1192/j.eurpsy.2025.1706

**Published:** 2025-08-26

**Authors:** S. Ben Aissa, N. Chaima, R. Khouloud, L. Amine, M. Wahid

**Affiliations:** 1 Psychiatry D, Razi Hospital, Mannouba, Tunisia

## Abstract

**Introduction:**

Global epidemiological research suggests that approximately 1% of the global population is affected by schizophrenia, with 2 to 3% experiencing obsessive-compulsive disorder (OCD). Additionally, a noteworthy proportion of individuals with schizophrenia also exhibit comorbid OCD. This overlap often complicates differential diagnosis, especially as many schizophrenia patients display obsessive and/or compulsive symptoms akin to those seen in OCD.

**Objectives:**

This literature review is designed to examine how frequently schizophrenia and OCD co-occur and to summarize the treatments reported in various studies.

**Methods:**

A comprehensive literature review was carried out focusing on the co-occurrence and treatment of schizophrenia and OCD. This review involved an extensive search of scientific publications, selecting articles pertinent to the subject from databases such as Scopus and PubMed using keywords like “schizophrenia and OCD” and “schizophrenia and OCD treatment,” yielding over 1500 articles from 1988 to 2024. Additional sources included Google Scholar and various grey literature sources. After applying specific exclusion criteria, 44 recent articles were selected that addressed the frequency of co-occurrence and the treatment modalities used for schizophrenia and OCD. These articles were categorized by country, frequency of co-occurrence, and treatment methods.

**Results:**

The review revealed that roughly 14.5% of individuals with schizophrenia also suffer from OCD. Among treatments, Clozapine was commonly associated with the worsening of OCD symptoms, while Haloperidol showed better tolerability. Other antipsychotic medications like Olanzapine, Risperidone, and Quetiapine were noted to either aggravate existing OCD symptoms or initiate the onset in some cases. In contrast, some antipsychotics, such as Amisulpride and Aripiprazole, helped reduce symptom severity. Non-pharmacological treatments, particularly Cognitive Behavioral Therapy (CBT), have proven effective in reducing symptom severity with favorable outcomes noted in numerous cases. The integration of pharmacological and psychotherapeutic strategies is generally recommended to maximize therapeutic outcomes, underscoring the potential of combined modalities in treating OCD.

**Conclusions:**

Our literature review investigated the prevalence of comorbidity between schizophrenia and obsessive-compulsive disorder (OCD), as well as the pharmacological and non-pharmacological treatments utilized. The significant overlap between these disorders highlights the complexity of managing patients with these co-occurring conditions, underscoring the need for systematic screenings and integrated treatment approaches.

**Disclosure of Interest:**

None Declared

